# Precisely Monomeric Linear RNAs of Viroids Belonging to *Pospiviroid* and *Hostuviroid* Genera Are Infectious Regardless of Transcription Initiation Site and 5′-Terminal Structure

**DOI:** 10.3390/cells10112971

**Published:** 2021-11-01

**Authors:** Tatsuji Hataya, Takashi Naoi

**Affiliations:** 1 Pathogen-Plant Interactions, Research Faculty of Agriculture, Hokkaido University, Kita 9, Nishi 9, Kita-ku, Sapporo 060-8589, Japan; 2 Pathogen-Plant Interactions, Graduate School of Agriculture, Hokkaido University, Kita 9, Nishi 9, Kita-ku, Sapporo 060-8589, Japan; naoi@res.agr.hokudai.ac.jp

**Keywords:** viroids, cDNA clone, transcripts, monomeric RNA, infectious RNA, 5′-terminus

## Abstract

Infectious dimeric RNA transcripts are a powerful tool for reverse genetic analyses in viroid studies. However, the construction of dimeric cDNA clones is laborious and time consuming, especially in mutational analyses by in vitro mutagenesis. In this study, we developed a system to synthesize a precisely monomeric linear RNA that could be transcribed in vitro directly from the cDNA clones of four viroid species. The cDNA clones were constructed such that RNA transcription was initiated at the guanine nucleotide of a predicted processing and ligation site in the viroid replication process. Although the transcribed RNAs were considered to possess 5′-triphosphate and 3′-hydroxyl termini, the RNA transcripts were infectious even without in vitro modifications. Additionally, infectivity was detected in the monomeric RNA transcripts, in which transcription was initiated at guanine nucleotides distinct from the predicted processing/ligation site. Moreover, monomeric viroid RNAs bearing 5′-monophosphate, 5′-hydroxyl, or 5′-capped termini were found to be infectious. Northern blot analysis of the pooled total RNA of the plants inoculated with the 5′-terminal modified RNA of potato spindle tuber viroid (PSTVd) indicated that maximum PSTVd accumulation occurred in plants with 5′-monophosphate RNA inoculation, followed by the plants with 5′-triphosphate RNA inoculation. Our system for synthesizing an infectious monomeric linear viroid RNA from a cDNA clone will facilitate mutational analyses by in vitro mutagenesis in viroid research.

## 1. Introduction

Viroids are the smallest infectious agents consisting solely of single-stranded (ss) circular RNA molecules ranging from 246 to 433 nucleotides (nt) in all species described untill now, including a new species named *Apple hammerhead viroid* [1]. Viroids do not have protein-coding capacity, and they replicate autonomously through a rolling-circle mechanism mediated by the enzymes of the host cells that they invade. Sometimes viroids may cause a disease in the host plants that they infect. Based on structural and biochemical features as well as the mode of replication, viroids can be classified into two families, *Pospiviroidae* and *Avsunviroidae*. Members of the family *Pospiviroidae* form rod-like secondary structures that can be divided into five structural and functional domains (terminal left, TL; pathogenicity, P; central, C or central conserved region, CCR; variable, V; and terminal right, TR). These viroids replicate within the nuclei of the infected cells through the asymmetric pathway of the rolling-circle mechanism. The viroid RNA invading the host cell is imported to the nucleus, where it redirects the host DNA-dependent RNA polymerase II (DdRP II) to accept the viroid RNA templates. Subsequently, a longer-than-unit-length RNA of complementary (−) polarity is synthesized from the circular RNA molecules of the viroid by DdRP II in the nucleoplasm. The DdRP II binds to the TL domain of the potato spindle tuber viroid (PSTVd) (+)-RNA strand and initiates its transcription from the nucleotide position U359 or C1 within the loop of the TL domain [2,3]. Thereafter, a longer-than-unit-length (+)-RNA strand is synthesized from the longer-than-unit-length (−)-RNA strand by the DdRP II within the nucleoplasm, and it is imported to the nucleolus. A splicing variant of eukaryotic transcription factor IIIA (TFIIIA-7ZF), which is present in the host cells, has been identified as an essential factor facilitating the DdRP II-mediated replication of PSTVd [4]. The longer-than-unit-length (+)-RNA strand is cleaved to a unit-length RNA molecule by a class III RNase enzyme [5], and this cleaved RNA molecule has 5′-phosphomonoester (5′-P) and 3′-hydroxyl (3′-OH) termini [6]. The viroid RNA also redirects the DNA ligase 1 of the host cell to function as an RNA ligase for the circularization of the linear viroid RNA molecule [7]. In contrast, members of the family *Avsunviroidae* such as peach latent mosaic viroid (PLMVd) and chrysanthemum chlorotic mottle viroid form branched secondary structures [8]. These viroids replicate within the chloroplasts of infected cells through the symmetric pathway of the rolling-circle mechanism; moreover, these viroid RNAs possess hammerhead ribozyme motifs that can catalyze self-cleavage in RNA strands of both the polarities. Hammerhead ribozymes generate monomeric linear RNA molecules bearing 5′-hydroxyl (5′-OH) and 2′,3′-cyclic phosphodiester (2′,3′ > P) termini [9]. The generated monomeric linear RNA is circularized mainly by the catalysis of a host tRNA ligase, as demonstrated in eggplant latent viroid [10], although RNA autocatalytic circularization has also been shown to occur in vitro in self-cleaving monomeric linear molecules of PLMVd [11].

Infectious cDNA clones form a powerful tool for reverse genetic analyses in studies on viroid replication, movement, and pathogenesis. Previously, studies on PSTVd [12,13,14] and hop stunt viroid (HSVd) [15,16,17] have demonstrated that cDNAs consisting of head-to-tail dimers of the entire viroid sequence and their RNA transcripts of (+) polarity are highly infectious to host plants. The redirection and subsequent binding of DdRP II to the viroid RNA template as well as the viroid complementary sequences for the synthesis of longer-than-unit-length (−) and (+) RNAs imply that both the RNA sequences can act as RNA promoters; in addition, the infectious nature of the viroid cDNA indicates that the viroid cDNA sequence can act as a DdRP II promoter. Moreover, the dimeric RNA transcripts derived from a dimeric viroid cDNA clone mimic the longer-than-unit-length replication intermediates; therefore, they can be processed into unit-length viroid RNA and circularized within the host plant cells. Infectivity studies using a series of deletion mutants of dimeric HSVd cDNA showed that the longer-than-unit-length cDNA molecules have the same infectivity as the dimeric cDNA molecules if they possess a 64-bp duplicated sequence containing the cDNA sequence of the upper CCR viroid domain [18]. In addition, certain studies have shown that an 11-nt sequence (GGATCCCCGGG) duplication derived from the cDNA of the upper CCR viroid sequence as well as the cloning vector significantly increases the infectivity of the cloned monomeric cDNAs of PSTVd and citrus exocortis viroid (CEVd) [13,19,20]; therefore, the processing site of the longer-than-unit-length viroid replication intermediates was presumed to exist within the abovementioned 11-nt sequence. Moreover, mutational analysis using the monomeric cDNA clone of CEVd suggested that the processing occurs at one of the three G nucleotides in positions 97‒99 in CEVd, which are corresponding to nucleotides 95‒97 in PSTVd [20]. Afterwards, S1 nuclease-mapping and primer extension analysis of the 5′ and 3′ termini of processing intermediates revealed that the cleavage and ligation site of PSTVd was located between G95 and G96 in the upper CCR viroid domain [21]. However, several monomeric CEVd RNA transcripts including non-viroid vector sequences at the 5′ and 3′ termini were demonstrated to be infectious to host plants regardless of the transcription initiation site in the CEVd sequence, except RNA transcripts derived from one cDNA clone [22]. Infectivity of monomeric viroid RNA transcripts including non-viroid vector sequences at the 5′ and 3′ termini has also been reported in chrysanthemum stunt viroid (CSVd) [23].

Meanwhile, Rigden and Rezaian demonstrated that an exact monomeric linear CEVd RNA without any non-viroid sequences that was transcribed in vitro with T7 RNA polymerase from the cDNA products amplified by reverse transcription and polymerase chain reaction (RT-PCR) was also infectious to host plants [24]. Similar to the aforementioned report [22], infectivity was observed for both the linear transcripts initiated at nucleotides G70 or G331 in the CEVd sequence, unlike the abovementioned processing site (G97-G98-G99) in the CEVd replication process. Although RNA molecules synthesized by in vitro transcription using T7 RNA polymerase possessed 5′-triphosphate (5′-ppp) and 3′-OH termini, modifications of the 5′-terminus to 5′-monophosphate (5′-p) or 5′-OH had little or no effect on the infectivity. Infectious transcripts synthesized from RT-PCR products do not require cloning in *Escherichia coli*. However, since viroid RNA extracted from infected plants may consist of quasispecies population due to spontaneous mutation, the RNAs transcribed using RT-PCR products amplified from these viroid RNAs may include heterogeneous sequence populations containing minor sequence variants [25]. Therefore, an exact monomeric linear RNA of some viroids was transcribed in vitro using the PCR products amplified from a cDNA clone that has been sequenced previously [26,27].

In this study, we developed an in vitro transcription system to synthesize an exact monomeric linear RNA transcript without any non-viroid sequence directly from the cDNA clone of PSTVd, CSVd, and tomato chlorotic dwarf viroid (TCDVd) in the genus *Pospiviroid* and HSVd in the genus *Hostuviroid*. Additionally, we re-evaluated the infectivity of monomeric viroid RNA transcripts possessing a different transcription initiation site and 5′-terminal structure.

## 2. Materials and Methods

### 2.1. Viroid Sources

Four viroid species were used in this study, namely, PSTVd-Intermediate (Int) (accession no. V01465, 359 nt) [28]; TCDVd-Canadian (Ca) isolate (accession no. AF162131, 360 nt) [29]; CSVd-TP2, which is a sequence variant with a uracil to cytosine substitution at nucleotide position 126 in a previously deposited sequence (accession no. AB006737, 354 nt) [30]; and HSVd-citV1(270G), which is a sequence variant with an adenine-to-guanine substitution at nucleotide position 270 in a previously determined sequence (accession no. X06718, 302 nt) [31].

### 2.2. Infectious Monomeric Viroid cDNA Clones

Monomeric cDNA sequences of PSTVd-Int and TCDVd-Ca were cloned into the BamHI site of a pUC9 vector. The screened cDNA clones were sequenced and confirmed to have a duplicated 11-nt sequence of 5′-GGATCCCCGGG-3′ in combination with the vector sequence.

### 2.3. Infectious Dimeric Viroid RNA Transcripts Derived from Dimeric cDNA Clones

A head-to-tail dimeric cDNA of PSTVd-Int was inserted downstream of the T7 class III phi6.5 promoter sequence created in a pUC119 vector (Takara Bio, Shiga, Japan) (Figure S1). A head-to-tail dimeric cDNA of HSVd-citV1(270G) was inserted downstream of the T7 promoter region of the pBlueScript II SK(-) vector (Agilent Technologies, Santa Clara, CA, USA) (Figure S1). The dimeric RNAs of PSTVd or HSVd were transcribed in vitro using T7 RNA polymerase. First, 5 µg of plasmid DNA containing dimeric cDNA of PSTVd or HSVd was linearized by restriction digestion with EcoRI or SpeI. Thereafter, it was incubated for 3 h at 37 °C in a 200 µL reaction mixture containing 40 mM Tris-HCl (pH 8.0), 8 mM MgCl_2_, 2 mM spermidine, 25 mM NaCl, 10 mM dithiothreitol, 100 U human placenta RNase inhibitor (FUJIFILM Wako Pure Chemical, Osaka, Japan), 0.5 mM of each rNTP, and 100 U T7 RNA polymerase (Invitrogen, Thermo Fisher Scientific, Waltham, MA, USA). Subsequently, the template plasmid DNA was digested by incubating the mixture with 5 U deoxyribonuclease (RT Grade; Nippon Gene, Toyama, Japan) for 15 min at 37 °C. Thereafter, the transcribed RNA was recovered by phenol-chloroform extraction, and the extracted RNAs were precipitated using 100 µL of 7.5 M ammonium acetate and 600 µL of ethanol. After allowing the mixture to rest for 30 min at –20 °C, RNA was collected by centrifugation at 16,000× *g* for 5 min. The obtained RNA precipitate was resuspended in 100 µL of 1 M ammonium acetate and again precipitated using 200 µL of ethanol to remove any unused rNTPs. Finally, the recovered RNA was resuspended in 20 µL of the RNA storage solution (Ambion, Thermo Fisher Scientific) consisting of 1 mM sodium citrate (pH 6.4) and quantified using a NanoDrop 1000 spectrophotometer (Thermo Fisher Scientific). Two quantities (1 and 0.2 µg) of each RNA sample were denatured with an equal volume of 2× RNA sample loading buffer (Norgen Biotek Corp., Thorold, ON, Canada) containing ethidium bromide (Et-Br) by incubation at 80 °C for 10 min, electrophoresed in a 1.2% denaturing agarose gel containing 2.2 M formaldehyde using 1× 3-(N-morpholino)propanesulfonic acid (MOPS) running buffer, and visualized under ultraviolet (UV) light at 312 nm.

### 2.4. In Vitro Synthesis of Monomeric Viroid RNA Transcripts

**Figure 1:**
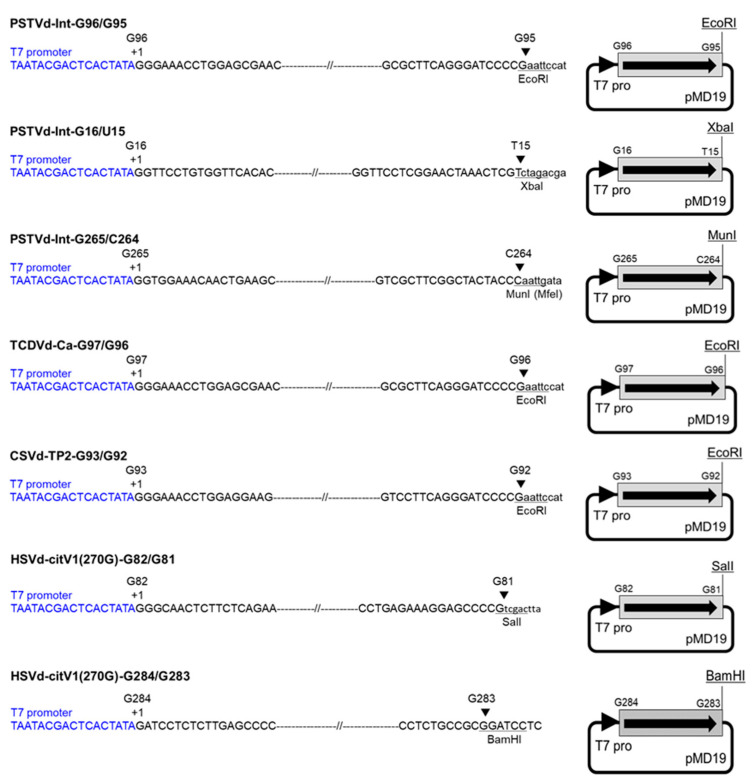
Construction of monomeric viroid cDNA clones to transcribe complete monomeric viroid RNAs without any non-viroid sequence. The cDNA clones were linearized by digestion with respective restriction enzymes, and monomeric RNAs were transcribed with T7 RNA polymerase. Each arrowhead indicates the T7 promoter sequence (T7 pro) shown in blue letters. Each arrow indicates the direction of viroid sequence shown with a gray rectangle. PSTVd: potato spindle tuber viroid; TCDVd: tomato chlorotic dwarf viroid; CSVd: chrysanthemum stunt viroid; HSVd: hop stunt viroid.

Monomeric cDNA was amplified by PCR from a previously prepared cDNA clone using *Ex Taq* (Takara Bio) or by RT-PCR from a viroid RNA using a forward primer containing T7 class III phi6.5 promoter sequence and a reverse primer containing a restriction enzyme site (Table S1). The forward primer was designed to initiate transcription at a guanine nucleotide, which is the 5′-terminal nucleotide of a determined or putative processing and ligation site in the upper CCR domain, namely, G96 of PSTVd, G97 of TCDVd, G93 of CSVd, and G82 of HSVd. Moreover, primers initiating transcription at G265 and G16 of PSTVd as well as G284 of HSVd were designed to compare the infectivity of RNA transcripts obtained from different transcription initiation sites (Table S1, Figure 1). The monomeric cDNA clones of PSTVd, TCDVd, and HSVd were used as templates for PCR. However, for CSVd, a low molecular weight RNA extracted from a CSVd-infected chrysanthemum plant was used as the template for RT-PCR with reverse transcriptase ReverTra Ace (Toyobo, Osaka, Japan) and *Ex Taq* (Takara Bio). The PCR products were purified from an agarose gel, ligated into a pMD19 T-vector (Takara Bio), and cloned into *E.* *coli* JM109. The sequences of cDNA clones were confirmed by sequencing. The cDNA clones were linearized by digestion with respective restriction enzymes (Figure 1), and the RNAs were transcribed with T7 RNA polymerase (Invitrogen), as described above.

### 2.5. Modification of 5′-Terminus of Monomeric Viroid RNA Transcripts

**Figure 2:**
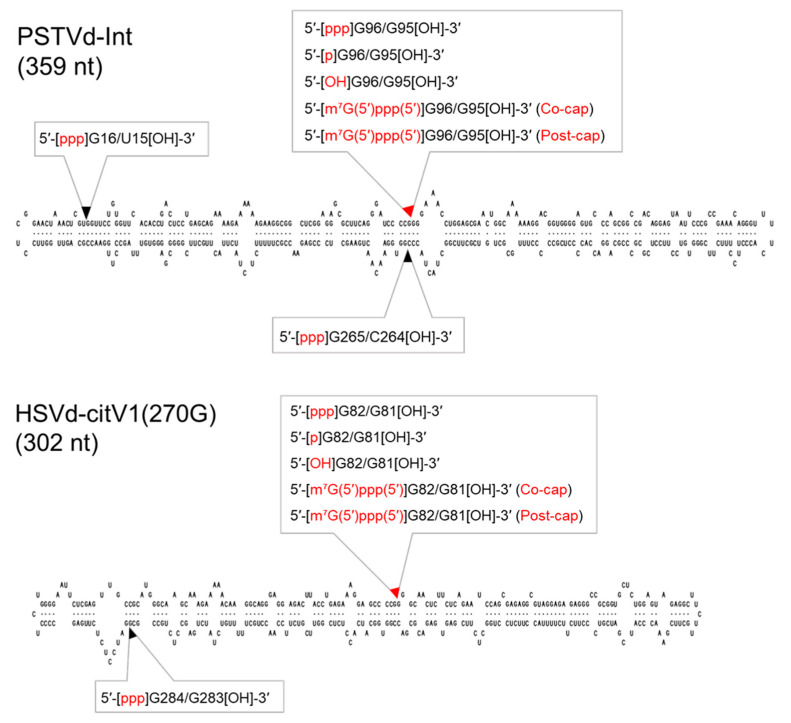
Transcription initiation sites and 5′-terminus modifications in viroid sequences to synthesize complete monomeric RNA transcripts. The constructions of monomeric viroid cDNA clones of potato spindle tuber viroid (PSTVd) and hop stunt viroid (HSVd) are described in Figure 1. Each arrowhead indicates a dividing position into a transcription initiation and termination site. Each red arrowhead indicates the putative processing/ligation site in the viroid replication process. [ppp]: triphosphate; [p]: monophosphate; [OH]: hydroxyl; [m^7^G(5′)ppp(5′)]: cap; Co-cap: co-transcriptional capping; post-cap: post-transcriptional capping.

The RNA transcripts that were synthesized in vitro using T7 RNA polymerase possessed 5′-ppp and 3′-OH termini. To evaluate the effect of the 5′-terminal structure on the infectivity of monomeric linear viroid RNA, the 5′-termini of PSTVd-Int-G96/G95, and HSVd-citV1(270G)-G82/G81 RNA transcripts were modified to 5′-p, 5′-OH, and 5′-cap [m^7^G(5′)ppp(5′)] (Figure 2).

Modification from 5′-ppp to 5′-p in the 5′-terminus of the RNA transcripts was conducted using RNA 5′ polyphosphatase (Epicentre, Lucigen, Middleton, WI, USA), as described in previous studies [7]. This included the incubation of 25 µg of the RNA samples for 1 h at 37 °C in a 100 µL reaction mixture containing 50 mM HEPES-KOH (pH 7.5), 100 mM NaCl, 1 mM EDTA, 0.1% 2-mercaptoethanol, 0.01% Triton X-100 (Epicentre), 80 U RNase inhibitor, and 20 U RNA 5′ polyphosphatase.

Modification from 5′-ppp to 5′-OH in the 5′-terminus of the RNA transcripts was conducted by dephosphorylation with Antarctic phosphatase (New England Biolabs, Ipswich, MA, USA). This included the incubation of 10 µg of the RNA samples for 1 h at 37 °C in a 100 µL reaction mixture containing 50 mM Bis-Tris-Propane HCl, 1 mM MgCl_2_, 0.1 mM ZnCl_2_, pH 6.0 (New England Biolabs), 40 U RNase inhibitor, and 50 U Antarctic phosphatase, followed by inactivation of Antarctic phosphatase via incubation for 5 min at 70 °C. Both of the modified RNA transcripts (5′-p and 5′-OH) were recovered by phenol-chloroform extraction and ethanol precipitation.

Co-transcriptional capping of the 5′-terminus was performed as described in previous studies [32]. First, 5 μg of linearized plasmid DNAs was incubated for 25 min at 37 °C in a 20 µL reaction mixture containing 40 mM Tris-HCl (pH 8.0); 25 mM NaCl; 8 mM MgCl_2_; 2 mM spermidine; 10 mM dithiothreitol; 2 mM each of ATP, UTP, and CTP; 0.2 mM GTP; 0.5 mM cap analog m^7^G(5′)ppp(5′)G (Invitrogen); 40 U of RNase inhibitor; and 50 U T7 RNA polymerase (Invitrogen). Subsequently, 2 μL of 20 mM GTP was added to the reaction mixture to a final concentration of 2 mM. After incubation for 50 min at 37 °C, the plasmid DNA template was digested with 5 U of RNase-free DNase I (Takara Bio) at 37 °C for 15 min.

Modification from 5′-ppp to 5′-cap (post-transcriptional capping) in the 5′-terminus of the RNA transcripts was conducted using the Vaccinia Capping System (New England Biolabs). For this, 10 µg of RNA samples were denatured at 65 °C for 5 min in 15 µL of ultrapure water, placed on ice for 5 min, and then incubated for 1 h at 37 °C in a 20 µL reaction mixture containing 1× capping buffer (New England Biolabs), 0.5 mM GTP, 0.1 mM SAM, and 1 µL Vaccinia Capping Enzyme. The co- and post-transcriptional capping reaction mixture volumes were then increased to 100 μL with ultrapure water; thereafter, 10 μL of a solution containing 5 M ammonium acetate and 0.1 M EDTA (pH 8.0) was added to the reaction mixture. The reaction products were purified by phenol-chloroform extraction, followed by precipitation with an equal volume of 2-propanol.

Recovered RNAs were resuspended in 11 µL of the RNA storage solution (Ambion) and quantified using a NanoDrop 1000 spectrophotometer (Thermo Fisher Scientific). Each RNA sample (0.5 µg) was checked by electrophoresis in a 1.2% denaturing agarose gel containing 2.2 M formaldehyde using 1× MOPS running buffer, as described above.

### 2.6. Inoculation to Plants and Evaluation of Infectivity of Inoculum Nucleic Acids

Plasmid DNAs or RNA transcripts were suspended in inoculation buffer (0.1 M Tris-HCl, pH 7.5; 10 mM EDTA, pH 7.5; 0.5 mg/mL bentonite). Thereafter, the inoculation suspension (1.25−1.5 µL) was rubbed onto carborundum (600 mesh)-dusted cotyledons (two cotyledons per plant) using a finger covered with a disposable latex finger cot. At least three seedlings at the cotyledon stage were used for each inoculation with DNA or RNA. The tomato (*Solanum lycopersicum*) cultivar “Rutgers” was inoculated with PSTVd and TCDVd; the tomato cultivar “Newskij” was inoculated with CSVd; and the cucumber (*Cucumis sativus*) cultivar “Suyo” was inoculated with HSVd. The inoculated plants were grown under greenhouse conditions of 24–28 °C (PSTVd, TCDVd, and HSVd) or in a growth chamber at 24 °C (CSVd). The infectivity of inoculum nucleic acids was evaluated by the extent of infection in inoculated plants. First, 5-fold serially diluted nucleic acids were used as an inoculum, and the inoculated plants were investigated by RT-PCR at 42- or 56-days post inoculation (dpi). Afterwards, the extent of infection in plants inoculated with a certain amount of RNA transcripts was investigated every 7 days to identify the approximate days required for the detection of a viroid by RT-PCR.

### 2.7. Detection of Viroids from Inoculated Plants by RT-PCR

A previously described method was used to extract total nucleic acids from approximately 50–100 mg upper uninoculated leaf tissue using potassium ethyl xanthogenate [33] with some modifications, as described by Hataya (manuscript submitted). The extracted nucleic acids were dissolved in 20 µL of ultrapure water, and cDNA was synthesized from 1−2 µL of the extracted total nucleic acid solution using reverse transcriptase ReverTra Ace (Toyobo) and random hexamer (Takara Bio) in a 10 µL reaction mixture. The viroid cDNA sequence was amplified from 1 µL of the synthesized cDNA by PCR in a 20 µL reaction mixture containing 10 mM Tris-HCl (pH 8.3), 50 mM KCl, 1.5 mM MgCl_2_, 0.2 mM of each dNTP, 0.2 µM of each target primer pair, and 0.5 U of Hot Start *Taq* DNA polymerase (New England Biolabs). The sequences of PSTVd, TCDVd, HSVd, and CSVd were amplified using their respective primer pairs PSTV-7P and PSTV-7M [34] (for PSTVd and TCDVd), HSV-8 and HSV-9 (for HSVd), and CSV-1P and CSV-1M (for CSVd) [30] (Table S1). Additionally, a primer pair of AtropaNad2.1a and AtropaNad2.2b was used for the amplification of an internal control [35] (Table S1). The PCR process included an initial denaturation at 95 °C for 3 min, followed by 35 cycles of denaturation at 95 °C for 20 s, annealing at 55 °C (PSTVd, TCDVd, and HSVd) or 61 °C (CSVd) for 30 s, and extension at 68 °C for 30 s, and a final extension at 68 °C for 5 min. Half of the RT-PCR products (10 µL) were electrophoresed in a 2% agarose gel using 0.5× TAE (20 mM Tris-acetate, 0.5 mM EDTA) running buffer and visualized under UV light after staining with Et-Br.

### 2.8. Northern Blot Hybridization

The accumulation of PSTVd in tomatoes inoculated with the different transcripts of PSTVd was compared by Northern blot hybridization. Total RNA was individually extracted using RNAiso Plus (Takara Bio) from the upper uninoculated leaves of five tomato plants 14 dpi. Subsequently, equal quantities of total RNA from the five plants were pooled and subjected to Northern blot hybridization, as described in previous studies [36]. The pooled total RNA (1 µg) was denatured at 65 °C for 10 min in the presence of 43.8% formamide and 5.2% formaldehyde. Subsequently, the RNA denaturation products were electrophoresed in 1.2% (*w*/*v*) agarose gel containing formaldehyde at a final concentration of 0.66 M in 1× MOPS buffer. The fractionated RNA was transferred from the agarose gel to a nylon membrane (Amercham Hybond-N, GE Healthcare, Chicago, Illinois, USA) by capillary transfer and hybridized with a DIG-labeled cRNA probe specific for PSTVd at 55 °C, as described in previous studies [37]. The hybridized RNA was detected using the DIG detection system and chemiluminescent substrate CDP-Star (Roche, Mannheim, Germany), and it was visualized using a luminescent image analyzer (ImageQuant LAS4000mini, Fujifilm, Tokyo, Japan).

## 3. Results

### 3.1. Precisely Monomeric Viroid RNA Transcripts Are Infectious to Host Plants

We inoculated plants with 2, 0.4, and 0.08 µg of plasmid DNA containing monomeric viroid cDNA along with the duplicated 11-nt sequence and monomeric or dimeric linear viroid RNA transcripts. Inoculated plants were assayed for infection at 42 dpi for PSTVd and TCDVd and 56 dpi for HSVd and CSVd. The infectivity of PSTVd-Int-G96/G95 and TCDVd-Ca-G97/G96 monomeric RNAs was higher than that of pUC9 inserted with monomeric cDNA of PSTVd and TCDVd, respectively. In addition, the infectivity of PSTVd-Int-G96/G95 and HSVd-citV1(270G)-G82/G81 monomeric RNAs was equal to or higher than that of PSTVd and HSVd dimeric RNAs, respectively (Table 1). Furthermore, infectivity was observed in PSTVd-Int-G265/C264, PSTVd-Int-G16/U15, and HSVd-citV1(270G)-G284/G283 monomeric RNA transcripts, which were initiated at the guanine nucleotide distinctly different from the processing/ligation site in the viroid replication process (Table 1). Therefore, all complete monomeric linear RNAs, including CSVd-TP2-G93/G92, examined in this study were infectious to host plants regardless of the transcription initiation site (Table 1).

### 3.2. Transcription Initiation at the Pcessing/Ligation Site Is Preferable but Not Necessary for the Production of Infectious Viroid RNA

The relationship between the putative processing/ligation site and the infectivity of viroid RNA transcripts was further investigated by comparing the time-dependent infection rate. In this experiment, monomeric (1 µg each) and dimeric (2 µg each) RNAs of PSTVd and HSVd were inoculated into “Rutgers” tomatoes and “Suyo” cucumbers, respectively. The reason is that one molecule of dimeric viroid RNA produces one molecule of monomeric viroid RNA by in vivo processing; therefore, 2 µg of dimeric viroid RNAs were inoculated, based on the number of monomeric RNA molecules that would be generated. Starting from 14 dpi, the extent of infection in the inoculated plants was investigated every seven days using RT-PCR. At 14 dpi, with respect to the infection rate of PSTVd, it was observed that PSTVd-Int-G96/G95 RNA had the maximum infectivity, followed by PSTV-Int-G16/U15, PSTVd-Int-2U and PSTVd-Int-G265/C264 RNAs. Moreover, the first infection by the PSTVd-Int-G265/C264 inoculum was confirmed seven days after the confirmation of infection with the other three inoculums. However, by 42 dpi, the infection with PSTVd-Int-G265/C264 RNA was eventually confirmed in all five tomato plants; this pattern showed a 14- or 21-day delay in comparison to the pattern of infectivity of PSTV-Int-G16/U15 and PSTVd-Int-2U RNAs or PSTVd-Int-G96/G95 RNA (Table 2). Incidentally, at 14 as well as 21 dpi, HSVd infection was confirmed only in plants inoculated with HSVd-citV1(270G)-G82/G81 RNA. However, by 35 dpi, the infection was eventually confirmed in all the cucumber plants inoculated with HSVd-citV1(270G)-G82/G81, HSVd-citV1(270G)-G284/G283, and HSVd-citV1-2U RNAs (Table 2).

### 3.3. Precisely Monomeric Viroid RNAs Are Infectious Regardless of 5′-Terminal Structures

To understand the infectivity of precisely monomeric viroid RNA transcripts possessing 5′-ppp, the 5′-ppp termini of PSTVd and HSVd monomeric RNA transcripts were modified to 5′-p, 5′-OH, or 5′-cap. Subsequently, each of these modified RNA transcripts was used as an inoculum at 0.4 µg per plant, except 2U PSTVd RNA, which was inoculated at 0.8 µg per plant. The 5′-capped transcripts were prepared using the following two procedures. Co-transcriptional capping is usually considered to produce a mixture of capped and uncapped (i.e., 5′-ppp) transcripts, whereas post-transcriptional capping produces nearly 100% capped transcripts. Starting from 14 dpi, the extent of infection in the inoculated plants was investigated every seven days using RT-PCR. Unexpectedly, at 21 dpi, a cucumber plant inoculated with 5′-OH monomeric HSVd RNA was the first to exhibit HSVd infection. Thereafter, at 28 dpi, three cucumber plants inoculated with 5′-ppp monomeric HSVd RNA and one cucumber plant inoculated with 5′-p monomeric HSVd RNA became positive for HSVd infection; however, cucumbers inoculated with co- and post-transcriptionally synthesized 5ʹ-capped HSVd RNA were still negative for HSVd infection. Eventually, by 35 dpi, all inoculated cucumbers were confirmed to be infected by HSVd (Table 3).

**Figure 3:**
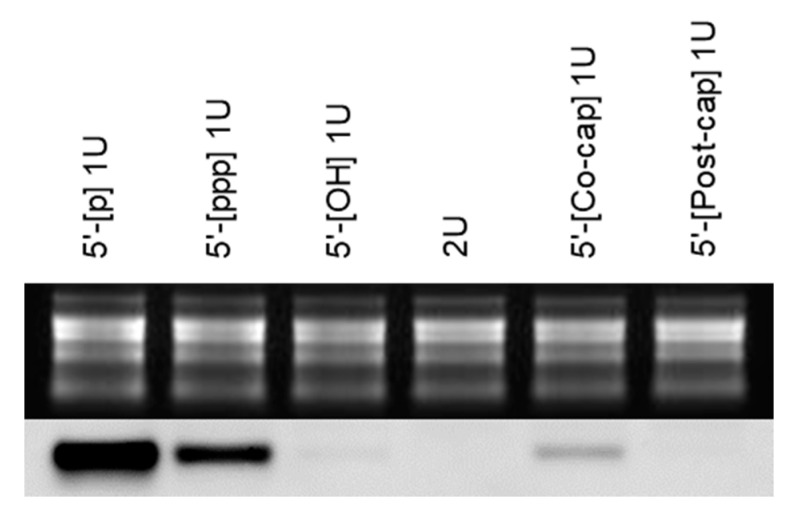
Northern blot hybridization using pooled total RNA extracted from tomato plants inoculated with the monomeric (1U) or dimeric (2U) RNA of potato spindle tuber viroid (PSTVd). Total RNA was individually extracted from five tomato plants, and equal quantities of total RNA were pooled and subjected to formaldehyde-denaturing agarose gel electrophoresis and visualized by Et-Br staining and UV irradiation (upper panel; loading control). Subsequently, RNA was transferred from the agarose gel to a nylon membrane and hybridized with a DIG-labeled cRNA probe specific for PSTVd (lower panel). Monomeric RNA possessed a different 5′-terminal structure; monophosphate (5′-[p] 1U), triphosphate (5′-[ppp] 1U), hydroxyl (5′-[OH] 1U), cap by co-transcriptional capping (5′-[co-cap] 1U), or cap by post-transcriptional capping (5′-[post-cap] 1U).

In the first inoculation experiment, all the tomato plants inoculated with the five different monomeric transcripts as well as one dimeric transcript of PSTVd were positive for PSTVd infection at 14 dpi, as detected by RT-PCR; these results were probably obtained due to the optimal plant growth conditions, such as abundant natural sunlight (Table 3). Since the first round of inoculation experiments with the different transcripts of HSVd and PSTVd did not show significant differences in infectivity, the second round of inoculation experiments was conducted only with PSTVd. However, in the second inoculation experiment, different infection rates were detected by RT-PCR among the six different inoculum transcripts at 14 dpi; these results were probably obtained due to the natural sunlight condition of being slightly inferior as compared with that in the first inoculation experiment. The extent of PSTVd accumulation in the different tomatoes at 14 dpi was compared by Northern blot hybridization. Even though positive PSTVd infection was confirmed by RT-PCR in all inoculated plants at 21 dpi, the PSTVd accumulation levels could be highly different among individual plants; therefore, we used pooled total RNA obtained by equally mixing the individual RNAs extracted from the five plants for the Northern blot hybridization. The accumulation of PSTVd was at a maximum in the plants inoculated with 5′-p RNA followed by that in the plants inoculated with 5′-ppp RNA, co-transcriptionally synthesized 5′-capped RNA, and 5′-OH RNA. However, PSTVd accumulation was not detected in the plants inoculated with post-transcriptionally synthesized 5′-capped RNA and dimeric RNA (Figure 3).

## 4. Discussion

We developed an in vitro transcription system for synthesizing a complete monomeric linear viroid RNA without any non-viroid sequence and with the ability to infect host cells from a cDNA clone. The infectious monomeric viroid RNA transcripts can serve as a powerful and useful tool for viroid studies. The infection efficiency of monomeric RNA transcripts initiated at the putative processing/ligation site was apparently higher than that of the pUC9 inserted with the corresponding monomeric cDNAs, and it was either equal to or more than that of the dimeric RNA transcripts (Table 1 and Table 2). However, at the same time, our results on the infectivity of monomeric linear viroid RNA transcripts raise new questions regarding the way in which (+)-circular viroid molecules can be produced from (+)-linear viroid RNA transcripts. Previous studies have reported that the linear forms of PSTVd and CSVd that accumulated naturally in infected tissues were as infectious as the circular forms [38,39,40]. Recently, it has been reported that a linear (+)-PSTVd would not serve as a template to synthesize a longer-than-unit-length (–)-PSTVd strand by DdRP II in the rolling circle replication process, and a circular (+)-PSTVd template is critical for the asymmetric rolling circle replication [41]. Based upon this report, the naturally occurring linear forms of viroid are assumed to serve after circularization in vivo as a template for the rolling circle replication. Therefore, the infectivity of monomeric linear viroid RNA transcripts in our results suggests that their circularization occurred in vivo after inoculation.

In addition to the transcripts mentioned above, monomeric RNA transcripts initiated at other guanine nucleotides distinct from the putative processing/ligation site were also found to be infectious (Table 1 and Table 2); these results are consistent with previously obtained reports [24,26]. However, analysis of the time-course of the infection rate suggests that the monomeric RNA transcripts initiated at the putative processing/ligation site are more efficient in infecting the host plant compared to the other monomeric RNA transcripts (Table 2). Monomeric linear PSTVd was demonstrated to be circularized in vitro between G96 and G95 but not between G265 and C264 or G16 and U15 by a tomato DNA ligase 1 even if it possesses 5′-P and 3′-OH termini. Therefore, it was considered that a specific terminal sequence and probably a specific conformation of RNA influenced the acceptance of a viroid RNA substrate by the tomato DNA ligase 1 [7]. However, the infectivity of PSTVd-Int-G265/C264 and PSTVd-Int-G16/U15 RNAs suggests that their RNAs circularized in vivo after inoculation. Hence, based upon our results, it may be suggested that the circularization partially occurred in vivo by the action of tomato DNA ligase 1 due to the differences in the in vitro and in vivo RNA conformation or by the activity of an RNA ligase completely different from tomato DNA ligase 1. 

In addition to the infectivity of monomeric PSTVd and HSVd RNA transcripts bearing the 5′-p terminus, infectivity was identified in the monomeric PSTVd and HSVd RNAs bearing the 5′-ppp, 5′-OH, and 5′-capped termini; these results are consistent with those from previous reports [23,24,26]. The time-course analysis of the infection rate using RT-PCR did not provide exact differences among the infectivities of the monomeric viroid RNAs bearing different 5′-structures (Table 3). However, the Northern blot hybridization analysis using pooled total RNA extracted from the plants inoculated with monomeric PSTVd-Int-G96/G95 RNAs bearing different 5′-terminal structures revealed the differences in the level of PSTVd accumulation (Figure 3). PSTVd accumulation was the maximum in plants inoculated with PSTVd-Int-G96/G95 RNA bearing the 5′-p terminus, suggesting that the linear viroid RNA bearing the 5′-p and 3′-OH termini was immediately circularized by the tomato DNA ligase 1 for replication, as previously reported [6,7].

Although a linear RNA bearing 5′-ppp and 3′-OH termini cannot be circularized directly, all the seven monomeric RNA transcripts of four viroids were infectious to host plants (Table 1, Table 2 and Table 3). Additionally, the second most abundant PSTVd accumulation was observed in plants inoculated with the 5′-ppp monomeric RNA (Figure 3). Based upon these results, it was assumed that the 5′-ppp terminus of monomeric RNA was immediately modified to 5′-p in vivo by a family of RNA polyphosphatases, which catalyze the removal of γ and β phosphates from a 5′-ppp RNA. Such RNA polyphosphatase activities have been previously identified in *Autographa californica* nuclear polyhedrosis virus BVP (baculovirus protein phosphatase) [42], *E. coli* RppH (RNA pyrophosphohydrolase; formerly known as NudH/YgdP) [43], human PIR1 (phosphatase that interacts with RNA-ribonucleoprotein complex 1) [44,45], and *Caenorhabditis elegans* PIR-1 [46]. However, similar RNA polyphosphatases have not yet been reported in plants; therefore, it is uncertain whether a plant RNA polyphosphatase is related to the infectivity of 5′-ppp monomeric linear viroid RNA. 

Previous studies have reported that the infectivity of co-transcriptionally capped monomeric CSVd RNA is the same as that of uncapped CSVd RNA, although the transcribed RNA includes a non-viroid sequence [23]. Incidentally, at 14 dpi, PSTVd accumulation was undetectable in plants inoculated with the post-transcriptionally capped monomeric RNA as compared to the PSTVd levels in plants inoculated with co-transcriptionally capped monomeric RNA (Figure 3). The co-transcriptionally capped RNA transcripts include a certain amount of uncapped 5′-ppp RNA; therefore, the infectivity of co-transcriptionally capped monomeric RNA may be due to the presence of 5′-ppp monomeric RNA. Eventually, all plants inoculated with the post-transcriptionally modified 5′-capped RNA were infected with PSTVd or HSVd; hence, the possibility that the 5′-capped monomeric viroid RNA was decapped in vivo with a low efficiency by certain decapping enzymes cannot be denied. 

Similar to the co-transcriptionally capped RNA products, the 5′-OH RNA products may include a small amount of 5′-ppp RNA; therefore, the infectivity of the 5′-OH RNA product may be due to the presence of the 5′-ppp RNA. Eventually, all plants inoculated with the 5′-OH RNA were infected with PSTVd or HSVd; therefore, it cannot be denied that there is a possibility that the 5′-OH termini of monomeric RNA transcripts may be phosphorylated with a low efficiency by an unknown mechanism in vivo. Another possibility is that the 3′-OH terminus may be phosphorylated to 3′-P, and subsequently, it may be converted to 2′,3′>P terminus. It has been previously reported that an RNA ligase purified from wheat germ is capable of ligating PSTVd linear molecules that have 5′-OH and 2′,3′>P termini, but it is incapable of ligating PSTVd linear molecules that have 5′-P and 3′-OH termini [47]. Based upon the studies carried out to date, the enzyme from wheat germ is a tRNA ligase, and the termini of PSTVd linear replication intermediates are 5′-P and 3′-OH. Meanwhile, Feldstein et al. had developed a ribozyme-mediated expression system to produce a precisely monomeric PSTVd RNA bearing 5′-OH and 2′,3′>P termini. The ribozyme-generated monomeric (‒) polarity PSTVd RNA was capable of circularization upon incubation with wheat germ extract. Additionally, the ribozyme-generated monomeric (+) polarity PSTVd RNA was found to be highly infectious to tomatoes [48]. These results suggest that the monomeric PSTVd RNAs bearing 5′-OH and 2′,3′>P termini are circularized in vivo by a cellular tRNA ligase, and they possess the ability to infect host plants. To the best of our knowledge, however, an enzyme that may phosphorylate 3′-OH to 3′-P termini has not yet been identified in living organisms, although RNA 3′-terminal phosphate cyclase is known to catalyze the ATP-dependent conversion of 3′-P terminus to 2′,3′>P in eukaryotes, bacteria, and archaea [49]. 

In conclusion, the precisely monomeric viroid RNA transcripts bearing 5′-ppp and 3′-OH are infectious to their host plants. Our in vitro transcription system of producing an infectious monomeric linear viroid RNA from a cDNA clone is convenient, and it will facilitate further viroid research since in vitro mutagenesis can be rapidly conducted by a simple procedure of inverse PCR, self-ligation, and cloning. Moreover, we recorded infectivity in monomeric RNA transcripts that were initiated at the guanine nucleotide identical to as well as distinct from the putative processing/ligation site, thereby suggesting the applicability of this system for studying viroids whose processing/ligation site is other than a guanine nucleotide.

## Figures and Tables

**Table 1:** Infectivity of monomeric or dimeric viroid RNA transcripts and plasmid DNA containing monomeric viroid cDNA.

Viroid	Inoculum Nucleic Acid	Inoculum Quantity (µg)			Assay Plant and Days Post-Inoculation (dpi)
		2	0.4	0.08	
Potato spindle tuber viroid (PSTVd)	1U RNA(G96/G95)	3/3 + 3/3 ^1^	3/3 + 3/3	3/3 + 3/3	“Rutgers,”^2^ 42 dpi
	1U RNA(G265/C264)	3/3	‒	‒	“Rutgers,” 42 dpi
	1U RNA(G16/U15)	3/3	‒	‒	“Rutgers,” 42 dpi
	2U RNA	3/3 + 3/3	3/3 + 3/3	0/3 + 3/3	“Rutgers,” 42 dpi
	1U cDNA-pUC9	1/3 + 3/3	1/3 + 2/3	0/3 + 0/3	“Rutgers,” 42 dpi
Tomato chlorotic dwarf viroid (TCDVd)	1U RNA(G97/G96)	5/5	4/5	3/4	“Rutgers,” 42 dpi
	1U cDNA-pUC9	4/5	1/5	0/4	“Rutgers,” 42 dpi
Chrysanthemum stunt viroid (CSVd)	1U RNA(G93/G92)	5/5	5/5	3/5	“Newskij,”^2^ 56 dpi
Hop stunt viroid (HSVd)	1U RNA(G82/G81)	3/3	3/3	3/3	“Suyo,”^2^ 56 dpi
	1U RNA(G284/G283)	3/3	3/3	3/3	“Suyo,” 56 dpi
	2U RNA	3/3	3/3	3/3	“Suyo,” 56 dpi

^1^ Number of plants infected/number of plants inoculated. A plus (+) indicates the result of an additional inoculation experiment. ^2^ “Rutgers” and “Newskij” are tomato cultivars, and “Suyo” is a cucumber cultivar.

**Table 2:** Time-course of the infection rate of plants inoculated with monomeric or dimeric viroid RNA transcripts.

Viroid	Inoculum Nucleic Acid ^1^	Days Post-Inoculation				
		14	21	28	35	42
Potato spindle tuber viroid (PSTVd)	1U RNA(G96/G95)	4/5 ^2^	5/5			
	1U RNA(G265/C264)	0/5	1/5	4/5	4/5	5/5
	1U RNA(G16/U15)	2/5	4/5	5/5		
	2U RNA	1/5	4/5	5/5		
Hop stunt viroid (HSVd)	1U RNA(G82/G81)	1/5	2/5	3/5	5/5	
	1U RNA(G284/G283)	0/5	0/5	2/5	5/5	
	2U RNA	0/4	0/4	3/4	4/4	

^1^ “Rutgers” tomatoes and “Suyo” cucumbers were inoculated with 1U (1 µg) or 2U (2 µg) RNA of PSTVd and HSVd, respectively. ^2^ Number of plants infected/number of plants inoculated.

**Table 3:** Time-course of the infection rate of plants inoculated with monomeric viroid RNA possessing a different 5′-terminus.

Viroid	Inoculum Nucleic Acid ^1^	Days Post-Inoculation			
		14	21	28	35
Potato spindle Tuber viroid (PSTVd)	5′-[ppp]G96/G95[OH]-3′ 1U RNA	(6/6), 5/5 ^2^			
	5′-[p]G96/G95[OH]-3′ 1U RNA	(6/6), 4/5	5/5		
	5′-[OH]G96/G95[OH]-3′ 1U RNA	(6/6), 3/5	5/5		
	5′-[m⁷G(5′)ppp(5′)]G96/G95[OH]-3′ (Co-cap) ^3^ 1U RNA	(6/6), 3/5	5/5		
	5′-[m⁷G(5′)ppp(5′)]G96/G95[OH]-3′ (Post-cap) ^3^ 1U RNA	(6/6), 4/5	5/5		
	2U RNA	(6/6), 1/5	5/5		
Hop stunt viroid (HSVd)	5′-[ppp]G82/G81[OH]-3′ 1U RNA	0/5	0/5	3/5	5/5
	5′-[p]G82/G81[OH]-3′ 1U RNA	0/5	0/5	1/5	5/5
	5′-[OH]G82/G81[OH]-3′ 1U RNA	0/5	1/5	2/5	5/5
	5′-[m⁷G(5′)ppp(5′)]G82/G81[OH]-3′ (Co-cap) 1U RNA	0/5	0/5	0/5	5/5
	5′-[m⁷G(5′)ppp(5′)]G82/G81[OH]-3′ (Post-cap) 1U RNA	0/5	0/5	0/5	5/5

^1^ “Rutgers” tomatoes and “Suyo” cucumbers were inoculated with 1U (0.4 µg) or 2U (0.8 µg) RNA of PSTVd and HSVd, respectively. ^2^ Number of plants infected/number of plants inoculated. The first and second results by PSTVd inoculation are shown in parentheses and non-parentheses, respectively. ^3^ “Co-cap” and “post-cap” indicate “co-transcriptionally capped” and “post-transcriptionally capped,” respectively.

## Data Availability

Not applicable.

## Supplementary Materials

The following are available online at https://www.mdpi.com/article/10.3390/cells10112971/s1, Figure S1: Construction of dimeric cDNA clones of potato spindle tuber viroid (PSTVd) and hop stunt viroid (HSVd); Table S1: List of primer pairs used in PCR.
