# Supplementary material for: Precisely Monomeric Linear RNAs of Viroids Belonging to Pospiviroid and Hostuviroid Genera Are Infectious Regardless of Transcription Initiation Site and 5′-Terminal Structure
Source: Cells. 2021 Nov 1;10(11):2971. doi: 10.3390/cells10112971 (PMC8616387; doi:10.3390/cells10112971)
Supplement: Supplementary file 1 [file cells-10-02971-s001.zip › cells-1398943-supplementary.pdf]

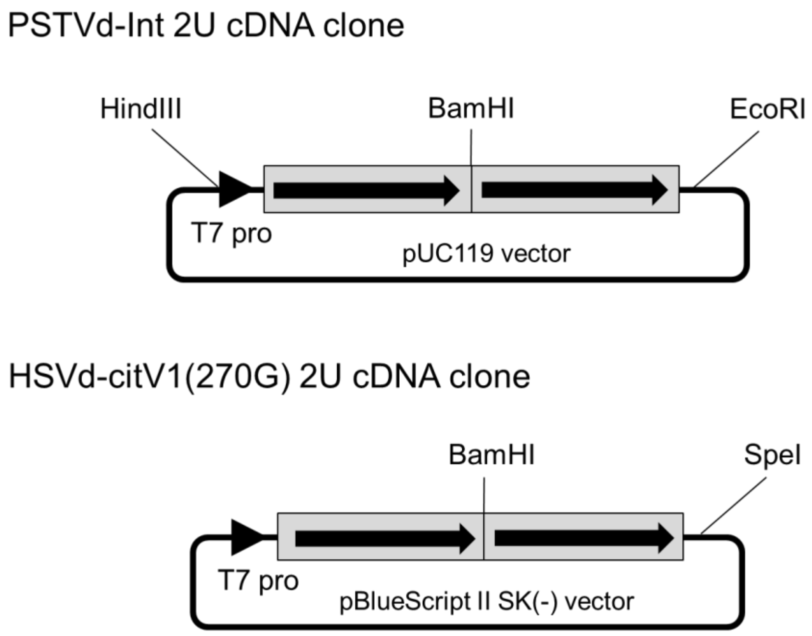

Figure S1. Construction of dimeric cDNA clones of potato spindle tuber viroid (PSTVd) and hop stunt viroid (HSVd). A gray rectangle indicates a unit-length viroid sequence. An arrow indicates the direction of viroid sequence. An arrowhead indicates T7 promoter sequence (T7 pro). EcoRI site in PSTVd clone and SpeI site in HSVd clone were used to linearize plasmids for the run-off transcription.

Table S1. List of primer pairs used in PCR

Primer name	Primer sequence 5′–3′	References
PSTV-7P	GGTTCCTGTGGTTCACACCTG	Hataya, 2009 [34]
PSTV-7M	GAACCAACTGCGGTTCCAAGG	
PSTV-13P	TAATACGACTCACTATAGGGAAACCTGGAGCGAAC	This study
PSTV-13M	atggaattCGGGGATCCCTGAAGCGC	PSTVd-Int-G96/G95
	EcoRI	TCDVd-Ca-G97/G96
PSTV-22P	TAATACGACTCACTATAGGTGGAAACAACTGAAGC	This study
PSTV-22M	tatcaattGGGTAGTAGCCGAAGCGAC	PSTVd-Int-G265/C264
	MunI (MfeI)	
PSTV-23P	TAATACGACTCACTATAGGTTCCTGTGGTTCACAC	This study
PSTV-23M	tcgtctagACGAGTTTAGTTCCGAGGAACC	PSTVd-Int-G16/U15
	XbaI	
HSV-8	CGCCTCTCGCTGGATTCTG	This study
HSV-9	GCGGTGCTCTGGAGTAGAG	
HSV-10P	TAATACGACTCACTATAGGGCAACTCTTCTCAGAA	This study
HSV-10M	taagtcgaCGGGGCTCCTTTCTCAGG	HSVd-citV1(270G)-
	SalI	G82/G81
HSV-11P	TAATACGACTCACTATAGATCCTCTCTTGAGCCCC	This study
PHSV268C	GAGGATCCGCGGCAGAGG	HSVd-citV1(270G)-
	BamHI	G284/G283
CSV-1P	CTTAGGACCCCACTCCTGCG	Li et al., 1997 [30];
CSV-1M	CCGCGATCTCGTCGGACTTC	Nakahara et al., 1999 [33]
CSV-3P	TAATACGACTCACTATAGGGAAACCTGGAGGAAG	This study
CSV-3M	atggaattCGGGGATCCCTGAAGGAC	CSVd-TP2-G93/G92
	EcoRI	
AtropaNad2.1a	GGACTCCTGACGTATACGAAGGATC	Thompson et al., 2003 [35];
AtropaNad2.2b	AGCAATGAGATTCCCCAATATCAT	Hataya, 2009 [34]

T7 promoter sequence is shown in blue letters in a forward primer sequence. Monomeric viroid RNA transcribed from the cDNA clone with T7 RNA polymerase is shown in red letters in "References." Restriction enzyme and its recognition sequence used to linearize a plasmid DNA for run-off transcription is shown in green letters in a reverse primer sequence. Lowercase letters in the reverse primer sequence indicate non-viroid sequence.
